# Domestic Medical Waste Management: An Assessment of Knowledge and Disposal Practices in the City of Tshwane Metropolitan Municipality

**DOI:** 10.3390/ijerph23020239

**Published:** 2026-02-14

**Authors:** Reneilwe Prudence Mariba, Matodzi Michael Mokoena, Thabiso John Morodi, Gomotsegang Fred Molelekwa

**Affiliations:** 1 Department of Environmental Health, Tshwane University of Technology, Private Bag X680, Pretoria Campus, Pretoria 0001, South Africa; mokoenamm1@tut.ac.za (M.M.M.); kunene.morodi@gmail.com (T.J.M.); 2 Regional Water and Environmental Sanitation Centre, Department of Civil Engineering, Kwame Nkrumah University of Science and Technology, Kumasi AK-039-5028, Ghana

**Keywords:** domestic medical waste, disposal practices, health risks, awareness

## Abstract

**Highlights:**

**Public health relevance—How does this work relate to a public health issue?**

- Domestic Medical Waste Management needs attention to prevent the spread of communicable diseases that could significantly impact the public health system in the City of Tshwane Metropolitan Municipality and in South Africa.
- Domestic medical waste is as infectious and hazardous as any other medical waste from a healthcare facility and needs to be managed properly. Failure to do so could lead to improper disposal thereof and could also lead to pollution of natural water resources, easy access of used needles and syringes by drug users, and sustained injuries (needle pricks) by waste pickers.

**Public health significance—Why is this work of significance to public health?**

- Provision of Domestic Medical Waste Management services by the City of Tshwane Metropolitan Municipality would ensure regular collection and proper disposal of DMW within the City of Tshwane Metropolitan Municipality, thus reducing and eliminating bad practices of DMW disposal such as dumping of DMW, pouring and flushing medicine in the toilet and putting DMW in the general municipal waste bin, with it thereby ending up at General Landfill sites.
- The proposed DMW Management Model would ensure integrated hazardous waste management within the City of Tshwane Metropolitan Municipality that will divert DMW from reaching General Landfills and reduce disease transmission, as well as educating residents in the City of Tshwane Metropolitan Municipality about proper management of DMW, thus saving the Municipality money that could have been spent on medical treatment.

**Public health implications—What are the key implications or messages for practitioners, policy makers and/or researchers in public health?**

- Domestic Medical Waste Management service must be an integral part of integrated waste management within the City of Tshwane Metropolitan Municipality.
- The Waste Management By-laws and the Integrated Development Plan (IDP) of the City of Tshwane Metropolitan Municipality must be reviewed to include DMWM service so that it can be budget for and be rendered accordingly.

**Abstract:**

The improper disposal of domestic medical waste (DMW) constitutes a significant public health and environmental concern; however, limited studies exist concerning DMW disposal practices in South Africa. This study evaluated the knowledge and practices involving the disposal of domestic medical waste (DMW) in the City of Tshwane Metropolitan Municipality. The study investigated common disposal methods, levels of awareness of appropriate techniques, and associated health risks. Data were collected using structured questionnaires (Annexure A) with closed-ended questions, administered both physically at shopping complexes and electronically via LinkedIn, WhatsApp, and email to eligible participants. Data analysis was conducted using the Statistical Package for the Social Sciences (SPSS) Version 29 and Microsoft Excel, with results presented in graphical form. Findings revealed that 78.3% of residents disposed of DMW in general waste bins, while 85.8% reported discarding medicine bottles in the same manner, and only 5.2% returned unused medications to pharmacies. The findings highlight gaps in awareness, infrastructure, and policy, necessitating comprehensive education programs, improved waste management services, and policy revisions to include DMW. A proposed model emphasizes education, community involvement, infrastructure enhancement, and ongoing policy evaluation to address these challenges. These efforts aim to reduce health risks, mitigate environmental impacts, and promote safe DMW disposal practices, safeguarding public health and creating a sustainable environment.

## 1. Introduction

The Constitution of South African, 1996 (Act 108 of 1996) [1], mandates that the state should provide proper health services, which includes domestic medical waste (DMW). However, currently, the state provides medical waste collection services at hospitals and clinics. On the other hand, municipalities are providing solid waste collection services to households, which indirectly encourages residents to dispose of their domestic medical waste (DMW) together with the municipal solid waste.

Medical waste refers to waste materials produced because of healthcare-related activities. These include diagnostic, treatment, or preventive procedures undertaken in hospitals, clinics, or at home [2]. Furthermore, this type of waste has been considered potentially harmful to humans and requires special attention. Similarly, Udofia et al. [3] alluded to the fact that there is a probable risk of Human Immunodeficiency Virus (HIV) transmission to a susceptible human host from needle prick injury. Subsequently, domestic medical waste is generated because of medical activities conducted in households. This waste includes but is not limited to: unused medication, medicine bottles, expired and used medication, as well as used bandages, syringes, and needles [4].

This study addresses a critical gap in the literature on domestic medical waste management, particularly in the context of South Africa. While significant research exists on healthcare facility-generated medical waste, domestic medical waste remains underexplored, despite its increasing prevalence and associated risks. With the rise in chronic diseases, self-medication, and home-based healthcare, households are becoming significant contributors of medical waste. However, there is limited understanding of how individuals manage this waste and the implications for public health and the environment.

The need for this research is underscored by Tshwane’s status as one of the top five districts with a high burden of disease in South Africa. With an HIV prevalence of 13.1% among people aged 15 to 49 years, the district is particularly vulnerable to the risks associated with improper DMW disposal. Chronic illnesses such as tuberculosis (TB), diabetes, hypertension, and arthritis are also prevalent in the region, contributing to increased pharmaceutical use and waste generation [5]. The management of DMW in Tshwane is critical to mitigating threats to human health and the environmental risks associated with improper management of domestic medical waste. The study was necessary to contribute to the body of knowledge with specific reference to knowledge, attitudes, and practices surrounding domestic medical waste management in the City of Tshwane Metropolitan Municipality. The study highlights the pressing need for effective policies, education campaigns, and disposal infrastructure tailored to the unique challenges of domestic medical waste management. In South Africa, where socio-economic disparities and infrastructural limitations pose additional hurdles, this research was particularly relevant in guiding targeted interventions.

The study aimed to determine the knowledge and practices of the residents of the City of Tshwane Metropolitan Municipality concerning domestic medical waste management, with specific focus on storage and disposal. The study objectives were to: determine domestic medical waste disposal practices, assess the knowledge of the community about the disposal of domestic medical waste, assess the health impact of domestic medical waste based on the knowledge and practices of the community, and to develop a model for the proper management of domestic medical waste within the City of Tshwane Metropolitan Municipality management practices.

## 2. Materials and Methods

This cross-sectional quantitative study was conducted in the City of Tshwane Metropolitan Municipality (November 2022–April 2023) among adult residents (≥18 years) selected using a calculated minimum sample size of 212 (Cochran’s formula). Inclusion criteria covered all adult residents within the City of Tshwane Metropolitan Municipality’s 7 regions, while residents under 18 or residing outside the municipality were excluded. Data was collected using a self-administered, structured questionnaire (Appendix A) informed by previous studies, piloted on 17 respondents within the City of Johannesburg Municipality, and refined for clarity. Distribution of questionnaires occurred physically at shopping centers and online platforms (LinkedIn, WhatsApp, email), introducing elements of convenience sampling, which limits representativeness and generalisability. Ethical approval was obtained from the TUT FCRE-SCI in Nov 2022, and informed consent was secured from participants. Data was analyzed using SPSS v29 and Excel, applying descriptive statistics, chi-square tests, and odds ratios, with significance set at *p* < 0.05.

## 3. Results

The results of this study are presented in Table 1, Table 2, Table 3 and Table 4. The results about the storage of medication by households are presented in Table 1. The results show that the most common place to keep medicine at home is the medicine cupboard, used by 108 respondents (50.9%). Second in place is the kitchen cupboard, used by 53 respondents (25%), followed by the fridge, used by 41 respondents (19.3%). Only a minority of respondents (4.7%) mentioned that the question did not apply to them, likely an indication that they did not keep medication in their homes. These results suggest that most households are aware that medication must be stored in a safe and proper place such as a medicine cupboard.

But the findings also reveal that a high percentage of households store medication in somewhat more unsuitable places, such as the fridge or kitchen cupboard. While refrigeration may be necessary for some drugs, poor storage in kitchen cabinets may expose medicines to heat, dampness, or contamination that may break them down. This underlines the importance of educating homes in proper storage of drugs to guarantee the efficacy of drugs and the safety of household members, especially children.

Table 2 presents findings on how the respondents dispose of used needles and syringes at home. The findings indicate a variety of disposal methods, including burning, flushing through the toilet, general waste containers, and medical waste containers. The table also indicates the participants who responded “not applicable,” denoting those who do not handle or generate such waste.

The findings show that a majority of the respondents (47.6%) dispose of empty needles and syringes into the general waste bin. The behavior is unsafe because it increases the risk of unintentional injuries and spread of infections to waste handlers and the community. Almost half of the participants (44.8%) reported that they do not have syringes or needles to discard and therefore selected “not applicable.” A smaller percentage (3.3%) reported burning the waste, and 1.4% reported flushing down the toilet, both of which are harmful and destructive to the environment.

Few of the respondents recognized the right way to dispose of the correct method through putting unused needles and syringes in a medical waste box or bin. These results indicate a weak gap in knowledge and accessibility of proper disposal methods for sharps. Incorrect disposal plays a big role in contributing to health and environmental risks, which indicates towards the need for better education as well as proper disposal facilities being accessible. Advertising and promoting the utilization of correct medical waste containers would minimize the risks of incorrect disposal methods.

Table 3 indicates the responses to how used medicine bottles are disposed of by participants at home. The findings in Table 3 also show that the majority of respondents (85.8%) dispose of used medicine bottles into the general waste bin. This implies that the majority of people do not use specialized or environmentally friendly methods of disposing of such bottles, which could be harmful through environmental pollution or accidental abuse. Fewer respondents (7.1%) said that the question did not pertain to them, and a meager 0.9% said they kept such bottles in the medicine cupboard.

On the other hand, few of the respondents did turn to safer or more environmentally conscious disposal practices. About 3.8% reported taking back empty medicine bottles to a pharmacy or hospital, as per safe disposal practices. Some 2.4% of them just left the bottles in their medicine cabinet, possibly for reusing or recycling. These outcomes highlight the need for increased awareness campaigns and easy disposal points to lead the public to adopt safer and more environmentally friendly practices.

Table 4 presents participants’ attitudes and perceptions towards domestic medical waste management. It reveals that the majority of the respondents agreed or strongly agreed that domestic medical waste be separated from general waste (94 agreed and 87 strongly agreed), a strong indicator of awareness of handling waste safely. A mere fraction (6 disagreed and 3 strongly disagreed) opposed segregation, but 22 were undecided. This indicates that a majority of the participants are aware of the health and environmental hazards posed by dumping medical waste along with general household trash.

In addition, the data also indicate very strong support for training and safe procedures. Combined, 114 respondents strongly agreed and 86 agreed that residents should be well educated in disposing of household medical waste, and this is a sign of concurrence that education is necessary within the community. Similarly, returning unwanted medication to a hospital or pharmacy was greatly supported, and 163 participants strongly agreed and 42 agreed. Lastly, an overwhelming majority of the respondents concurred (79) or strongly concurred (82) that medicine should be stored out of children’s reach, though 17 did not agree and 8 strongly did not agree, indicating that while there is extreme awareness, homes might under-exaggerate the risk.

## 4. Discussion

The increasing global use of medications necessitates a critical evaluation of awareness levels regarding their environmental and public health impacts. Research, such as the study by Rani et al. [6], highlights a concerning lack of knowledge about safe disposal practices for expired and unused medications. Findings reveal poor awareness and attitudes among consumers, further emphasizing the urgent need for education and policy interventions in this area. A strong correlation exists between regional differences and the presence of expired medications in households (*p*-value = 0.000), suggesting that certain areas face more significant challenges related to the storage and disposal of expired medications. These challenges could stem from limited access to disposal services or varying levels of awareness among residents. Notably in this study, many respondents store medications (1.42–4.25%) in kitchen cupboards and (1.87–4.25%) in the refrigerators, indicating a lack of knowledge about safe storage practices.

Education on proper disposal methods has the potential to influence how residents manage their medical waste. The close relationship between the belief that residents should be trained on DMW disposal practices and medication storage practices (*p*-value = 0.08) highlights the importance of awareness campaigns. However, inconsistencies in applying safe disposal methods persist. For example, while respondents acknowledge the risks (n = 86) of used syringes, many (86%) still dispose of them improperly put literature. This could be attributed to a lack of limited resources, inadequate infrastructure, and awareness [7]. A notable finding was the lack of a significant association between the belief that syringes can be disposed of with general waste and overall medication storage practices (*p*-value = 0.000), emphasizing gaps in understanding and application of safe disposal practices. This suggests that while residents may follow safe storage practices, their understanding of proper disposal remains insufficient. Addressing these gaps requires more than just awareness, it necessitates targeted education programs, clearer domestic medical waste disposal guidelines, and improved accessibility to proper disposal facilities.

The study revealed significant challenges in the disposal of needles and syringes. Nearly half (47.6%) of respondents disposed of used needless in general waste bins, while smaller percentages (3.3%) burned it and (1.4%) flushed them down the toilet These methods pose severe risks, including needlestick injuries, environmental contamination, and potential harm to aquatic ecosystems. For example, flushing medications like ibuprofen and diclofenac into water systems not only pollutes aquatic environments but also causes bioaccumulation, threatening marine life and, subsequently, human health through the food chain. To validate this, according to Vumazonke et al. [8], pharmaceutical waste in the environment poses a concern, as some of these substances are persistent and can enter the human body through the food chain or drinking water. Munzhelele et al. [9], alluded to the fact that pharmaceutical waste including antibiotics, antiretrovirals, and hormones is increasingly detected in water bodies, raising concerns due to its harmful effects on human health and ecosystems. The study highlighted that common contaminants such as ampicillin, penicillin, amoxicillin, diclofenac, paracetamol, vancomycin, sulphathiazole, carbamazepine, efavirenz, aspirin, and ibuprofen persist in surface water, groundwater, and wastewater.

These pollutants contribute to serious health and environmental issues, including drug resistance, endocrine disruption, infertility, cancer, and reduced growth in plants and animals. The sources of pharmaceutical contamination include sewage discharge, landfill leachate, improper waste disposal, and stormwater runoff. In South Africa, rural areas rely heavily on groundwater and urban areas depend on surface water, pharmaceutical pollution poses significant health and environmental risks [9]. Positively a minority (2.8%) of respondents used appropriate disposal methods, such as using medical waste boxes for disposal, however this points to a broader lack of access to safe disposal practices. Furthermore, 44.8% of respondents reported “not applicable” when asked about syringe disposal. This highlights the need for targeted education programs to improve awareness and safe practices. Local authorities play a crucial role in addressing these challenges. A significant association was found between the belief that authorities should provide waste disposal services and residents’ syringe disposal practices (*p*-value = 0.07). This suggests that while residents recognize the need for proper disposal infrastructure, many rely on the government’s intervention to facilitate safe practices. Current gaps in waste management services result in improper disposal practices, such as the disposal of used medicine bottles in general waste bins (5.66–18.4% across the City of Tshwane regions). The findings of this study also indicated a common practice of expired medications being disposed of in household drains, and toilets, thus contributing to environmental pollution. Furthermore, medication is stored in kitchen cupboards, which is viewed as an improper practice of storing medication further intensifies health risks, as unsecured storage increases the likelihood of accidental ingestion or injuries among children. Additionally, the absence of systems to track medication usage, coupled with residents (patients’) tendencies to discontinue prescribed treatments prematurely, leads to the accumulation of unused medications, increasing the burden of pharmaceutical waste.

Human excretions containing pharmaceutical residues should also be classified and managed as hazardous waste to mitigate environmental contamination. Schenck et al. [10], conducted a study in Samora Machel township (in the city of Johannesburg) and highlighted that municipalities provide waste management services only to formal settlements, as a result, informal settlements manage their waste. The inadequate provision of waste services leads to improper disposal methods, such as dumping, burning, and burying waste, including absorbent hygiene products (AHPs)/diapers [10]. Furthermore, the study indicated that the disposal practices have harmful effects on the environment, as well as human health. The widespread practice of disposing of DMW in general waste bins or water systems perpetuates the misconception that these methods are acceptable. Addressing these issues requires comprehensive educational campaigns, improved access to disposal services, and robust policy frameworks. Such interventions will not only safeguard public health but also minimize the environmental impact of domestic medical waste.

Improper management and disposal of domestic medical waste is a global concern that demands urgent attention. Numerous studies have explored this issue, with most focusing on disposing of unused or expired medicine and sharps waste. For instance, Chung and Brooks. [11] surveyed 1865 respondents across seven sites, revealing that 75% of households stored medicines, with each household averaging 138.4 g of stored medicines. This accumulation poses risks, particularly to children, due to accidental consumption or poisoning. Huang et al. [12] highlighted that medicine storage for emergencies or chronic treatment is prevalent globally, particularly in developing countries, where irrational storage and disposal practices are common. Studies like those by Bashaar et al. [13], Mitiku et al. [14] and Begum et al. [15] consistently show that many households store medicines, often unsafely, increasing risks of accidental poisoning and misuse. Moreover, Althagafi et al. [16] found that 64.4% of respondents stored medicines in refrigerators, while Azad et al. [17] reported that nearly half stored their medicines in bedrooms. These global practices reveal widespread issues with storing and disposing of medicines, which necessitates the development of effective domestic medical waste management strategies, such as collection services or medicine return programs. In Africa, managing domestic medical waste poses unique challenges, particularly regarding the disposal and storage of unused and expired medicines. Studies like those by Aboagye & Kyei [18] in Ghana, Kampamba et al. [19] in Zambia, and Marwa et al. [20] in Tanzania reveal widespread home storage of unused medicines, with rates ranging from 35% to 96% among respondents. Disposal practices often involve placing unused medicines in general waste bins, as seen in studies by Amoabeng et al. [21] in Ghana, where 70.8% of respondents disposed of expired medicines in general waste. Similar patterns are evident in Ethiopia, and Nigeria, where respondents commonly dispose of medicines in household bins or drains, despite environmental and health risks. The waste management hierarchy concept, “from cradle to grave,” is relevant for Africa, emphasizing the need for proper storage, disposal, and treatment. This requires public awareness campaigns, accessible disposal facilities, and stricter enforcement of medical waste management policies. In South Africa, limited research exists on domestic medical waste management compared to healthcare facility-generated waste. However, existing studies highlight significant trends. Magagula et al. [22] found that 51% of Johannesburg households stored medicines in medicine boxes, while Okonkwo Ihebe [23] reported similar practices in Cape Town, with 39% of respondents storing unused medicines. Disposal practices in South Africa mirror global trends, with many respondents disposing of medicines in general waste bins or down the drains, as Magagula et al. [22] and Hangulu & Akintola [24] noted. Such practices contribute to environmental issues, including wastewater pollution and eutrophication, as Mokoena et al. [25] observed in regions like Diepsloot. The disposal of needles in general waste bin can lead to major health risks and injuries, especially to children, waste pickers and homeless people in South Africa. Homeless drug addicts are also at risk of sharing used needles when administering the drugs through a method just known as “Bluetooth”. This unsafe practice, as described by Sparks [26] based on Johannesburg experience, involves two processes, first, injecting someone with a drug (i.e., Nyaope, which is a harmful mixture of heroin, antiretroviral HIV medication, crushed glass, and rat poi-son), second, quickly drawing blood from that person and immediately share it with the next user [26]. The worst-case scenario of this act is transmission of HIV/AIDS among the users [26]. Similarly, Tharoor [27], also reported that bluetoothing is a rare practice in South Africa where individuals inject the blood of someone who has consumed drugs like heroin, or antiretroviral medicine. The writer also discussed the health risks (HIV/AIDS and other illnesses that can be transmitted through bluetoothing).

**Figure 1:**
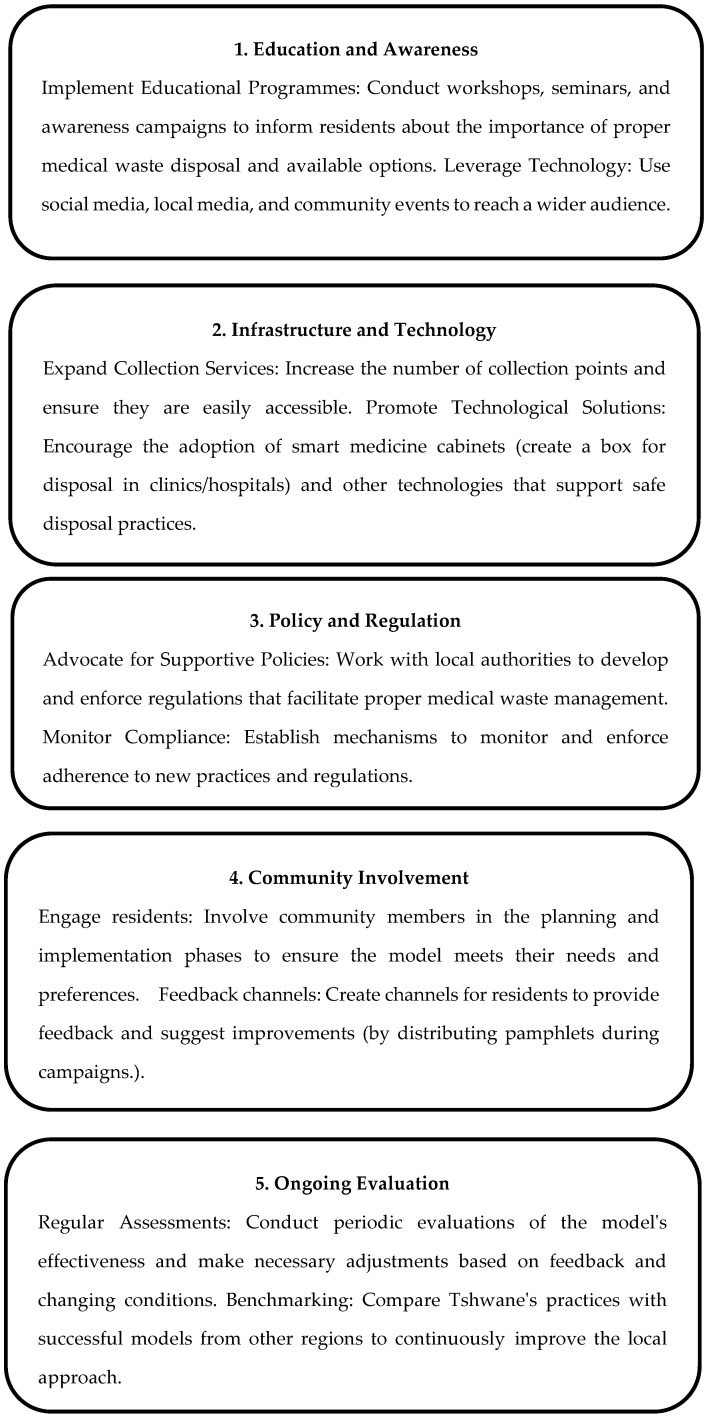
Proposed model for managing domestic medical waste.

The results have also shown that more (78.3%) respondents disposing of domestic medical waste (DMW) in general waste bins and water gives the impression that it is okay. Despite these challenges, the study also identified good practices. A small percentage (2.8%) of respondents returned unused medications to pharmacies or hospitals, a behavior that should be encouraged and scaled up. Given the results presented in the study and the available literature regarding DMW disposal and management, this study proposes a model to better manage DMW, (Figure 1).

The proposed model contains five aspects that will lead to proper disposal practices. Emphasis should be on education and awareness through the implementation of educational programs through workshops, seminars, and campaigns to promote proper medical waste disposal. Additionally, leveraging social media platforms such as Facebook, Instagram, Twitter, and TikTok, along with collaborations with influencers, can significantly enhance outreach and engagement, ensuring that proper disposal practices reach a wider audience effectively. Another important aspect is the infrastructure and technology which can be achieved through the extension of collection services by municipalities by using existing facilities such as return-back programs; e.g., expired/unused medicine and needles. Social media platforms or databases can also be used to request for returning medication, this will help with reducing accumulation of unused or expired medications in households as various return back scheduled dates can be followed in the platforms for communication. The third aspect is the policy and regulation as existing By-laws should be reviewed to cover DMW services. Department of Environmental Affairs and Tourism [28] must be changed to include DMW. Similarly, the National Waste Management Strategies and the Integrated Waste Management Plans must also be reviewed to include DMW services. For community involvement aspect, active community participation and the involvement must be fostered in the development of the model for rendering of DMW services in South Africa. Lastly on the aspect of ongoing evaluation, the community should also be part of the monitoring, evaluation and review of the model/policies, by-laws and plans for the rendering of DMW services.

## 5. Conclusions

This study demonstrates that domestic medical waste (DMW) in Tshwane is predominantly disposed of in unsafe practices, with most residents disposing of medicines and sharps in general waste bins. Such practices reflect limited awareness, inadequate infrastructure, and the absence of structured collection or take-back programs, posing risks to public health and the environment.

The study was limited to households in the seven regions of the City of Tshwane Metropolitan Municipality, included only participants aged 18 years and above, and excluded healthcare facilities and pharmacies. COVID-19 restrictions also constrained data collection, necessitating reliance on online responses. These factors, combined with convenience sampling and reliance on self-reported data, limit the generalizability of the findings.

Future research should examine longitudinal behavioral patterns, assess the effectiveness of existing and proposed policy interventions, and evaluate innovative disposal approaches, such as community collection points or pharmacy-based return programs, to enhance safe disposal practices. Overall, the findings highlight an urgent need for improved education, stronger integration of municipal services, and policy reform to ensure the safe management of DMW, thereby minimizing health risks and safeguarding the environment. In addition, future studies should investigate the feasibility of extending medical waste collection services to households in South Africa, as well as assess the extent and effectiveness of advocacy efforts promoting responsible medical waste management.

The proposed model for managing domestic medical waste (DMW) emphasizes the need for comprehensive domestic waste management systems, more especially, collection and disposal systems. Drawing lessons from practices during the COVID-19 pandemic, where red plastic bags were provided for DMW disposal and later collected to be disposed of from the healthcare facilities, similar approaches can be adopted for domestic medical waste collection, especially pharmaceutical waste. The provision of waste containers or boxes at clinics, allows residents to return unused medications during their consultations. Additionally, Community Healthcare Workers (CHCW), who already provide home-based care, can play a pivotal role by collecting pharmaceutical waste during their routine visits and drop it off at clinics, hospitals, or any designated facility.

To mitigate the environmental and health risks of disposable diapers, especially for children and adults on chronic medication, public awareness campaigns should educate caregivers on proper disposal methods. Waste management services must be improved to ensure the safe collection and disposal of used diapers, preventing fecal contamination and disease transmission. Promoting sustainable alternatives, such as biodegradable diapers or modern cloth options, can reduce long-term pollution. Additionally, community-based diaper collection programs should be established to prevent open dumping and facilitate proper waste management.

These measures are particularly applicable to domestic medical waste, as used diapers from chronically ill patients may contain pharmaceutical residues and pathogens, thereby increase the risk of environmental contamination and public health hazards if not managed appropriately. There is a need to extend DMW disposal systems beyond healthcare facilities to include households. In addition, the Healthcare Risk Waste Management Strategy should be reviewed and revised to explicitly incorporate domestic medical waste management. This process should also assess the current state of supporting infrastructure. Furthermore, healthcare risk waste management should be formally integrated into the Integrated Development Plan (IDP).

## Figures and Tables

**Table 1:** Storage of medication by households.

How Do You Store Medication in the Household?	Frequency (n)	Percentage (%)
Fridge	41	19%
Kitchen cupboard	53	25%
Medicine cupboard	108	51%
Not applicable	10	5%
Total	212	100

**Table 2:** Disposal of syringes/needles by households.

How Do You Dispose of Unused Needles/Syringes?	Frequency (n)	Percentage (%)
Burn the waste	7	3%
Flush down the toilet	3	1%
General waste bin	101	48%
Medical waste box/container	6	3%
Not applicable	95	45%
Total	212	100%

**Table 3:** Disposal of medicine bottles by households.

How Do You Dispose of Used Medicine Bottles?	Frequency (n)	Percentage (%)
General waste bin	182	85.8
Medicine cupboard	2	0.9
Not applicable	15	7.1
Return to pharmacy/hospital	5	2.4
Store inside the medicine cupboard	8	3.8
Total	212	100

**Table 4:** Knowledge of proper disposal of domestic medical waste by households.

	Agree	Disagree	Neither Agree nor Disagree	Strongly Agree	Strongly Disagree
Domestic medical waste must be segregated from general waste	94	6	22	87	3
Residents must be trained on the proper disposal of domestic medical waste	86	5	3	114	4
Returning unused medication to the pharmacy or hospital is a healthy and safe decision to make	42	1	2	163	4
Stored medication must be out of reach of children	79	17	26	82	8

## Data Availability

The original contributions presented in this study are included in the article. Further inquiries can be directed to the corresponding authors.

## Abbreviations

AIDS	Acquired Immune Deficiency Syndrome
HIV	Human Immuno-deficiency Virus
CHCW	Community HealthCare Workers
DMW	Domestic Medical Waste
IDP	Integrated Development Plan

## Appendix A

**Table A1:** Questionnaire.

Sample No……………….				Date………/…………/202.			
Section 1							
Name of Region	1	2	3	4	5	6	7
Respondents position in the household	Head of household	Spouse	Son	Daughter	Other relatives		
Age	18–25	26–35	36–45	46–55	56+		
Gender	Male	Female	Other				
Section 2							
Items in the household	Expired medication	Unused medication	Used cotton wool	Syringes/needles	Swabs	Medical waste bag/plastic	Medical waste box/container
Where do you get medical waste items?	Clinic	Pharmacy	Hospital	Traditional Healer	Other (specify)		
Section 3							
How do you dispose of used medicine bottles?	General Waste Bin	Medical Cupboard	Return to Pharmacy/hospital	Not Applicable			
How do you dispose of used needles/syringes?	Brun the waste	Flush down the toilet	General Waste Bin	Medical waste	Not Applicable		
